# Layered material platform for surface plasmon resonance biosensing

**DOI:** 10.1038/s41598-019-56105-7

**Published:** 2019-12-30

**Authors:** F. Wu, P. A. Thomas, V. G. Kravets, H. O. Arola, M. Soikkeli, K. Iljin, G. Kim, M. Kim, H. S. Shin, D. V. Andreeva, C. Neumann, M. Küllmer, A. Turchanin, D. De Fazio, O. Balci, V. Babenko, B. Luo, I. Goykhman, S. Hofmann, A. C. Ferrari, K. S. Novoselov, A. N. Grigorenko

**Affiliations:** 1 School of Physics and Astronomy, University of Manchester, Manchester, M13 9PL UK; 2 Key Laboratory for Non-Equilibrium Synthesis and Modulation of Condensed Matter (Ministry of Education), School of Science, Xi’an Jiaotong University, Xi’an, Shaanxi 710049 China; 3 VTT Technical Research Centre of Finland Ltd., P.O. Box 1000, FI-02044 VTT Espoo, Finland; 4 Department of Energy Engineering, Ulsan National Institute of Science & Technology (UNIST), Ulsan, 44919 Republic of Korea; 5 Department of Chemistry, Ulsan National Institute of Science & Technology (UNIST), Ulsan, 44919 Republic of Korea; 6 Low Dimensional Carbon Material Center, Ulsan National Institute of Science & Technology (UNIST), Ulsan, 44919 Republic of Korea; 7 Department of Materials Science and Engineering, National University of Singapore, Singapore, 117575 Singapore; 8 Institute of Physical Chemistry, Friedrich Schiller University Jena, Lessingstraße 10, 07743 Jena, Germany; 9 Cambridge Graphene Centre, University of Cambridge, Cambridge, CB3 OFA UK; 10 Chongqing 2D Materials Institute, Liangjiang New Area, Chongqing, 400714 China

**Keywords:** Optical properties and devices, Imaging and sensing

## Abstract

Plasmonic biosensing has emerged as the most sensitive label-free technique to detect various molecular species in solutions and has already proved crucial in drug discovery, food safety and studies of bio-reactions. This technique relies on surface plasmon resonances in ~50 nm metallic films and the possibility to functionalize the surface of the metal in order to achieve selectivity. At the same time, most metals corrode in bio-solutions, which reduces the quality factor and darkness of plasmonic resonances and thus the sensitivity. Furthermore, functionalization itself might have a detrimental effect on the quality of the surface, also reducing sensitivity. Here we demonstrate that the use of graphene and other layered materials for passivation and functionalization broadens the range of metals which can be used for plasmonic biosensing and increases the sensitivity by 3-4 orders of magnitude, as it guarantees stability of a metal in liquid and preserves the plasmonic resonances under biofunctionalization. We use this approach to detect low molecular weight HT-2 toxins (crucial for food safety), achieving phase sensitivity~0.5 fg/mL, three orders of magnitude higher than previously reported. This proves that layered materials provide a new platform for surface plasmon resonance biosensing, paving the way for compact biosensors for point of care testing.

## Introduction

The unique properties of graphene and related layered materials (GRMs) are promising for applications in many areas^1–3^. In biology and healthcare, their chemical, electronic and optical properties offer exciting opportunities. GRMs have the ultimate surface to volume ratio, leading to strong interactions with biological systems. In addition, graphene’s conductivity is strongly influenced by interaction with ad-atoms yielding electrical single atom detection^4^. Several groups reported graphene applications in biosensing^4–7^. However, the use of graphene electronic devices for bio-detection is not straightforward, due to the complex graphene surface chemistry and electronic noise^6^.

An alternative approach is based on hybrid technologies where a layered material (LM) serves as a bio-functional surface, while detection is performed using conventional label-free optical transducers^6–9^. References ^10,11^ reported that a combination of graphene with surface plasmon resonance (SPR) technology can provide such a hybrid. Being impenetrable to most atoms and ions^1^, GRMs can protect reactive metals (Cu, Ag, etc.) for a long (around a year) time in both air and water environments^11^. Thus, graphene protected Cu can undergo functionalization and various nanofabrication procedure without degradation of its properties^11^. SPR chips based on graphene protected cu show dark plasmon resonances (~0.01% reflection at resonance minimum) of high quality factors (>10), with 3 orders of magnitude better phase sensitivity than that of conventional Au films, due to better morphology of the deposited Cu^11^. Generally, LMs allow protection of the metal surface from the environment as well enabling the functionalization required to achieve selectivity. By bringing the functional sensing groups very close to the sensing metal surface, GRM monolayers offer very good protection against corrosion^11^, removing the need to functionalize the metal surface (which might damage the plasmonic properties of the metal) by being themselves bio-functionalized. This broadens the possible avenues for bio-functionalization and opens new opportunities for SPR biosensing, which, at present, lacks the sensitivity required to detect small (~1 fg/mL) concentrations of drugs, vitamins, antigens and viruses, as these can be deadly or pathogenic even in this ultra-low quantities.

Here, we present a layered material platform for SPR biosensing. We address three critical steps: i) robust protocols for metal protection using various LMs, ii) functionalization protocols for LMs on metallic surfaces, iii) bio-detection protocols which can be used with graphene protected metal. We note that ref. ^11^ introduced the use of graphene protection of the plasmonic properties of metals. However, steps ii) and iii) were not previously systematically discussed in literature and are generic to any type of hybrid biosensing. As a demonstration, we fabricate ultrasensitive SPR sensor functionalized with an anti-HT-2 toxin Fab fragment (HT2-10) and detected HT-2 toxin (~424 Da) with an amplitude detection limit ~1 pg/mL, 3 orders of magnitude better than currently available methods^12,13^ and a phase sensitivity limit ~0.5 fg/mL, which is comparable with label techniques. This paves the way to LM-based label-free SPR biosensing of small bio-objects at ultra-low concentrations. It is important to stress that enhancement of SPR sensitivity observed in our work comes from using phase sensitive methods instead of conventional amplitude detection^14^. However, a very high level of sensitivity enhancement (several orders of magnitude) comes from extreme resonance darkness, which can be achieved and conserved in water environment only in LM protected metal films. In addition, LM functionalization (in contrast to conventional Au functionalization) provides new avenues in achieving selectivity of bio-detection.

## Results

### Graphene-protected SPR biosensor

**Figure 1:**
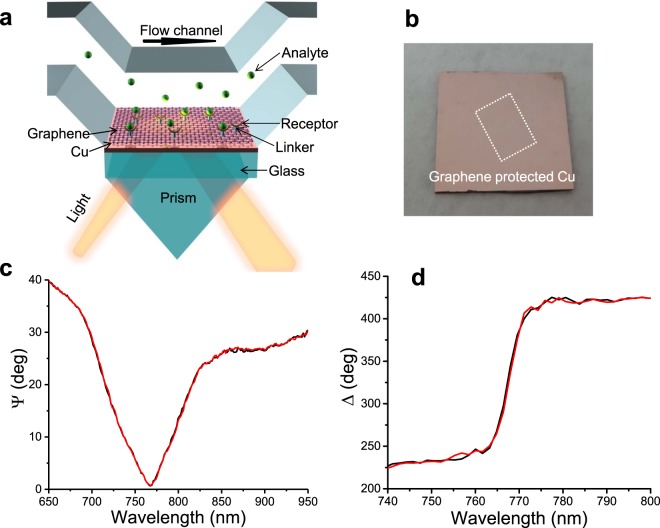
Graphene-protected Cu SPR biosensor. (**a**) Schematic diagram of flow cell for SLG-protected Cu SPR biosensing. (**b**) Image of a typical graphene-protected Cu SPR sensor chip. (**c**,**d)** Stability of the SPR in buffer solution: The change of Cu SPR ellipsometric parameters (amplitude ψ and phase Δ) of the sensor chip after pumping buffer solution for 15 minutes (the black and red curves). The sensor chip is pre-functionalized with receptor. The buffer solution is 1 mM NaP buffer (pH 7.3).

SPR biosensing^15^ exploits the excitation of surface plasmon polaritons in thin (~50 nm) metal films. The reaction between bio-receptors and bio-analytes modifies the surface plasmon polariton propagation and the optical reflection from a SPR chip changes significantly^15^. State-of-the-art SPR sensors provide selectivity, strong light confinement, and allow one to study bio-processes dynamics^16^. However, they lack sensitivity to measure small (<1000 Da) molecules and bio-objects, and cannot compete with label based technologies and be accessible for general use. LM protected SPR biosensors could solve this problem by providing unprecedented phase sensitivity to binding events^17,18^. Figure 1a is a schematic diagram of a flow cell for SPR biosensing. Graphene protected metal thin films (Cu is taken as an example, more combinations of layered LMs oxides and metals are described in Supplementary Materials) in which high (<1 fg/mL) sensitivity is achieved due to high quality plasmon resonances (see Methods). The surface plasmon polaritons in the metal film are excited in attenuated total reflection (ATR) geometry^19–21^. In the biosensing experiments, the analyte solutions are pumped into the flow cell. The binding of analyte to receptor on the sensor surface changes the local refractive index. These changes are monitored by the Cu SPR response, by measuring the ellipsometric amplitude ψ and phase Δ, see Methods.

Figure 1b shows a typical graphene protected Cu SPR sensor chip. The dotted rectangular frame indicates the edge of the transferred single layer graphene (SLG). Unlike SLG-protected Cu, unprotected Cu oxidizes after exposure to water after ~30 minutes. Typical SPR curves for SLG procted Cu samples in NaP buffer solution are shown in Fig. 1c,d. They confirm excellent SPR repeatability in graphene protected Cu after continuous buffer pumping, suggesting full protection of Cu by SLG. In these experiments, the SLG on the sensor chip is pre-functionalized with receptors before being fixed in the flow cell. This indicates that neither specific nor non-specific binding happen at a detectable level on the chip surface in presence of buffer.

The low values of reflection ~0.01% observed at the SPR minimum (the darkness of SPR resonances) are necessary to achieve high values of phase sensitivity^18^. These values cannot be achieved for standard Au SPR chips designed for biosensing due to intrinsic roughness of evaporated and sputtered Au films^16^. At the same time, extremely dark SPR resonances can be observed in fresh Cu and Ag films. However, they deteriorate fast in water (and even air) environment^11^. Only combination of graphene (or other GRMs) with suitably chosen metal films^11^ can unlock (in principle unlimited) phase SPR sensitivity of bio-detection.

### Protection of plasmonic properties of metals using layered materials

#### Effect of graphene transfer protocols on SPR quality

**Figure 2:**
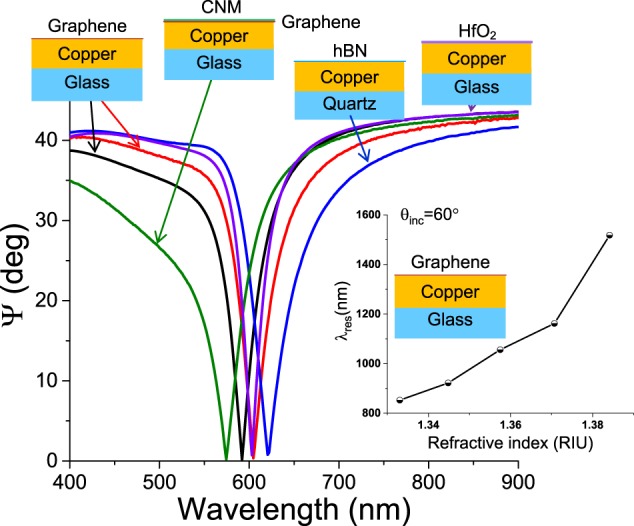
Protection of plasmonic properties of metals using various materials. SPR curves measured in Cu protected by SLG. Red and black curves correspond to P1, P1; blue, hBN, purple 10 nm HfO_2_, green SLG and Carbon Nanomembrane (see Methods). All curves are measured in the attenuated total reflection geometry in air. The Cu thickness for all samples is 43.5 nm. The inset plots the spectral position of the SPR minimum as a function of the refractive index of the medium contacting the SLG protected Cu structure.

Generic transfer protocols for SLG protection of metals were described in ref. ^11^. We first check the effect of different protocols on the graphene protected Cu plasmonic properties. To this end, we fabricate SPR chips based on SLG protected Cu using wet transfer of chemical vapour deposition (CVD) graphene as described in Supplementary Information, Protocol 1, P1. The corresponding SPR spectrum of SLG protected Cu in air is shown in Fig. 2 (red curve), where it is compared to the SPR spectrum from the samples obtained using a different wet transfer protocol, Protocol 2, P2, as described in Supplementary (black curve). For both protocols, we observe very deep (~0.01% reflection at resonance minimum) and high quality factor (>10) durable (for a period of a year) resonances, suggesting that the procedure is robust and peculiarities of graphene transfer do not affect the possibility of using graphene protected Cu in point-of-care testing. We stress that this is not a given, since transfer protocols can have massive influence on the final SLG properties, as discussed in ref. ^22^. The right inset of Fig. 2 shows the dependence of resonance wavelength on refractive index of a water-glycerol mixture for SPR based on SLG protected Cu. The response is ~10^4^ nm/RIU, where RIU is refractive index unit. This is in line with the sensitivity of standard Au SPR chips^16^. At the same time, the darkness of the resonances (i.e, the reflection value at the resonance minimum) in SLG protected Cu chips is two orders of magnitude better than Au, yielding enhanced phase sensitivity.

#### Refinement of graphene-based protection with the help of an additional protection

While the transfer protocol does not affect the SPR graphene protected Cu resonances quality, the durability of samples depends strongly on the SLG quality, as the life-time of SPR graphene protected Cu chips made from SLG with large amount of defects and pinholes decreases from months to weeks in water. The life-time can be improved significantly by depositing an additional ultrathin (~10 nm) layer of oxide (e.g., HfO_2_, Al_2_O_3_, etc.) before SLG transfer, or by transfer of a Carbon Nanomembrane (CNM) on SLG. A CNM is a ~1 nm thick material prepared by low energy electron induced crosslinking of aromatic self-assembled monolayers^23^. Due to its dielectric nature, CNMs can preserve SLG electronic properties via encapsulation^24^, as detailed in Supplementary Information. One could also make use of multiple SLGs to improve durability^11^. Figure 2 plots SPR resonances for Cu SPR samples protected by 10 nm HfO_2_ oxide with SLG on top, as well as CNM encapsulating SLG protected Cu with life-time >1 year (blue and green curves, respectively). Supplementary information reports measurements of SPR sensitivity for Ag samples protected by oxides and SLG, reaching ~2·10^4^ nm/RIU. Note that there are no known protocols for HfO_2_ bio-functionalization, while CNM does not always show good adhesion to metals.

#### Protection of plasmonic properties of metals by other layered materials

Protection of metals by other LMs can yield additional benefits. E.g., being similar to SLG in crystalline structure, hBN could be a viable alternative to SLG for metal protection. At the same time, the hBN dielectric nature does not suppress the metal SPR. Figure 2 shows a deep and high quality SPR curve measured with SPR chips made from Cu and hBN, as detailed in Supplementary Information. The durability of Cu protection offered by hBN is 10 times lower than SLG, probably due to different adhesion and hydrophobic properties of the two materials.

#### Direct growth of graphene for plasmonic protection

SLG can be directly grown on Cu^25^. CNMs can also be directly prepared on metal substrates by vapor deposition of aromatic molecules and subsequent electron irradiation in vacuum^26^. Hence, one could avoid transfer and grow SLG and CNMs directly on the SPR Cu chip. We thus fabricate SPR Cu substrates using electron beam deposition with excellent plasmonic resonances^11^, and then grow SLG and CNMs, as detailed in Supplementary Information. Supplementary Fig. 2 shows that the quality of the plasmonic resonances after growth is poor. This is probably due to the change of Cu morphology during growth, which is performed at 1000 °C, close to the Cu melting point ~1085 °C^25^.

Thus, wet transfer of CVD SLG on SPR structures results in robust SPR chips with excellent plasmonic resonances (darkness of 0.01%) with long (~year) life-time in air and water. An additional thin (~10 nm) layer of oxide deposited on the metal prior to transfer allows one to tune the resonance spectral position and add further protection. Alternatively, CNMs encapsulating SLG enhance the life-time and allows the use of a different bio-functionalized strategy based on amino groups^24^.

### Functionalization of graphene

The success of Au based SPR biosensors^15^ is also due to the progress in Au surface functionalization^27–30^, often based on well-developed self-assembly and chemistry of thiol alkane layers^29^. While phase SLG protected Cu SPR biosensors have 6 orders of magnitude higher sensitivity than amplitude Au based SPR (~0.2 fg/mm^2^ measured using phase detection with SLG hydrogenation^11^ versus ~1 pg/mm^2^ measured in amplitude detection^15^), there is a question whether functionalization of GRMs compatible with SPR biosensing is possible.

The SLG surface can be functionalized by either covalent or non-covalent bonding^31–35^. The first step consists in introducing COOH or NH_2_ endgroups for the attachment of bio-receptors. When both bio-receptor and bio-analyte are small (<1000 Da) molecules, this can be achieved during a biosensing protocol, as detailed below for HT-2 toxin detection. For larger molecules (fragments of DNA, aptamers, etc.), COOH or NH_2_ terminal groups should be attached to SLG, with a density that would stop the binding events from overlapping (grafting^36,37^). Since both Cu and SLG are good conductors, the covalent functionalization of graphene protected Cu can be performed using electrochemistry^38^. The protocol and outcome of functionalization are described in Supplementary Information. The grafting density is controlled by the Faraday law, and hence can be manipulated by changing the time of grafting. Functionalized GRMs can be activated, modified through chemical reactions to other ending, while preserving excellent SPR characteristics.

### Detection of HT-2 toxin by graphene-protected Cu SPR biosensor

We consider the detection of small (<1000 Da) molecules where conventional Au SPR lacks sensitivity^16^. To demonstrate the potential of LM protected SPR chips, we detect the HT-2 toxin with functionalized SLG protected Cu. Direct comparison to the state-of-the-art SPR detection based on Au is reported in Supplementary Information.

HT-2 is a fungal metabolite belonging to a family of mycotoxins, referred to as the trichothecenes, with a molecular weight ~424 Da, see ref. ^12,13^. It is the main metabolite of T-2 mycotoxin^12^. Both toxins are produced by moulds that grows on improperly stored grains^12^. HT-2 can cause acute or chronic intoxication of humans and animals^39,40^. The ability to detect low quantities of HT-2 is therefore of great interest for food safety and it can be performed by conventional SPR techniques based on Au with limited sensitivity (~ng/mL) as reported in ref. ^14^.

**Figure 3:**
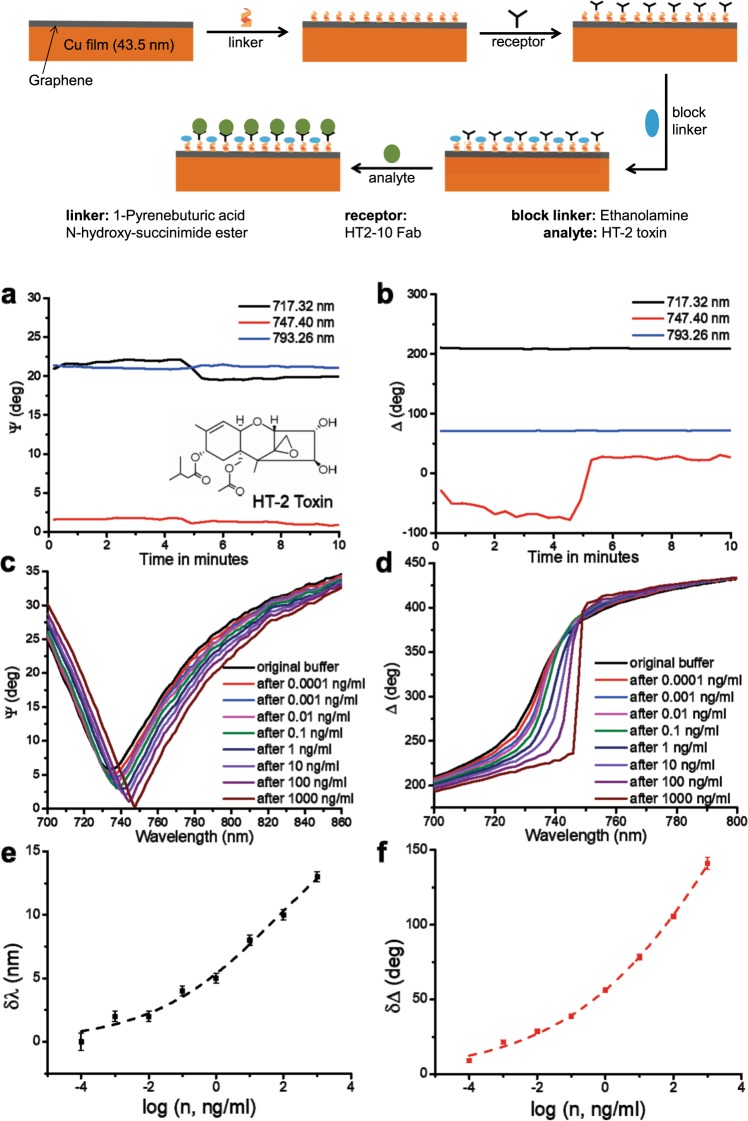
Graphene protected Cu SPR biosensing of HT-2. **(a**,**b**) Ellipsometric parameters ψ (amplitude) and Δ (phase) of the SPR curve of functionalized SLG protected Cu chips at 717.32, 747.40 and 793.26 nm, as a function of time when the sensor chip reacts with HT-2. The pumping time of PBS is~4.5 mins. The inset in (**a**) shows the molecular structure of HT-2. (**c**,**d**) SPR spectral curves after reacting with different concentrations of HT-2 in PBS. (**e**,**f**) The shift of resonant wavelength **(**δλ) for ψ and the change of phase **(**δΔ) as a function of logarithm of concentration, *n*, of HT-2. The dark dashed line shows the sigmoidal fit of δλ as a function of log(*n*), and the red dashed line is the same for δΔ, giving *n*_*H*_ = 0.2 ± 0.01 and *K*_*H*_ > 1 µg/mL. The top inset schematically describes the protocol of SLG functionalization for HT-2 bio-sensing.

The protocol for SLG functionalization for HT-2 detection with graphene protected Cu SPR is shown in the top inset of Fig. 3. This method was previously studied and characterized in detail^41^ and it has earlier been used for successful Fab fragment immobilization on graphene^42,43^. It yields the areal number density of active biorecognition sites at the level of ~3·10^11^ 1/cm^2^. A sensor chip is pre-functionalized with linker and receptor (see Fig. 3 and Methods for details). The linker (1-Pyrenebuturic acid N-hydroxy-succinimide ester) has a phenyl ring in the chemical structure that binds to SLG by π–π stacking. An amide group on each receptor (HT2-10 Fab fragment) binds to the NHS ester on the linker. Ethanolamine is used to block linkers not reacted with receptors to prevent non-specific binding. After rinsing in de-ionized water, the sensor chip is fixed on the flow channel and phosphate buffered saline (PBS) with and without HT-2 is alternately pumped into the flow cell. When toxins bind to the surface within the range of the electric surface plasmon polariton field they modify the local refractive index near the surface of the metal and thereby change the SPR properties (resonance wavelength and light phase) directly monitored by ellipsometry.

Figure 3a,b plot the change of the ellipsometric parameters (ψ and Δ) at 717.32, 747.40, and 793.26 nm, as a function of time in PBS containing~1 pg/mL HT-2. For SLG protected Cu film functionalized with HT2-10 Fab fragment the resonance maximum is at 747 nm at an incident angle of 59^0^. Therefore, the greatest phase sensitivity occurs at 747.40 nm, while the phase at the other wavelengths remains approximately constant. After pumping ~1 pg/mL of HT-2 solution into the flow channel there is no substantial change in amplitude (ψ) at all wavelengths. The phase has >100 degrees jump at 747.40 nm during the first 4.5 minutes, while there is almost no change at the other two wavelengths. The large phase change at 747.40 nm shows that phase measurements can provide higher sensitivity than amplitude measurements. Figure 3e,f show the evolution of resonant wavelength and light phase, due to the change of refractive index induced by adding different concentrations of HT-2 superimposed with sigmoidal modelling of the curves (the dashed lines).

## Discussion

**Figure 4:**
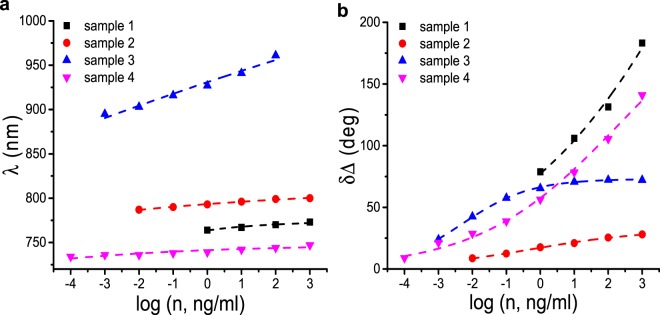
Sensitivity detection of SLG-protected Cu SPR biosensors. (**a**) change of spectral position Ψ_min_(λ) as a function of concentration (n) of HT-2. (**b**) corresponding change (jump) of phase **(**δΔ**)** as a function of n. Dashed lines are sigmoidal fits of λ_min_ and δΔ.

The change of resonant wavelength shift and phase change with increasing toxin concentration is described well by a sigmoidal function^44^. This fit has its physical origins in the Langmuir isotherm model^45,46^ and its derivatives (such as the Hill equation^47^) which describe the adsorption of molecules on to a surface. Originally, the Hill equation provided a description of the binding of ligand to receptors sites on proteins at equilibrium, as a function of ligand concentration, *c*^47^. It can be written as $f(c)=\frac{1}{{(\frac{{k}_{H}}{c})}^{{n}_{H}}+1}$, where *f* is the fraction of sites occupied by ligands, *k*_*H*_ is the ligand concentration at which half of the available receptor sites are occupied, and *n*_*H*_ is the Hill coefficient, describing cooperativity of ligand binding^47^. Positive cooperativity, *n*_*H*_ > 1, implies that the binding of a ligand increases the binding affinity of the neighboring sites^47^. Negative cooperativity, *n*_*H*_ < 1, occurs when the binding of a ligand decreases the binding affinity of the neighboring sites^47^. In first approximation, we can consider the shift of resonance wavelength and light phase at resonance to be proportional to the site occupancy number *f*(*c*), taken in the sigmoidal form. The sigmoidal fits provide very good approximation to the measured data, see Fig. 3e,f, where the fits are plotted as dotted lines. Figures 3 and 4 indicate that the detection limit of amplitude measurements is ~1 pg/mL, 3 orders of magnitude higher than in refs. ^12,13^. Figure 3d plots changes in phase due to binding with increasing HT-2 concentration. Near the resonant wavelength we observe a big phase jump (~200 degrees). The relations between phase (δΔ) and HT-2 concentration are shown in Fig. 3f. The phase change (~8.9 degrees) after testing on 1 pg/mL HT-2 reveals an ultrasensitive detection limit ~0.5 fg/mL for an experimental ellipsometer phase resolution ~0.05 degrees. (The improvement over conventional amplitude sensitivity comes about from the darkness of SLG protected Cu resonances and enhanced stability of phase measurements^48,49^). This limit could be pushed down to ~0.1 fg/mL in a dedicated phase setup capable of a phase resolution of 0.01 degrees^50^, 1000 times more sensitive than amplitude measurements. The sensitivity of commercial Au SPR for HT-2 is >1 ng/mL^12,14,51^. To check this, we performed measurements of HT-2 with Au Biacore chips on a Biacore T-200, using the same protocol and achieved sensitivity~1 ng/mL, as detailed in Supplementary Information.

We note that the SPR properties of SLG protected metals in water significantly depend on metal thickness^16^, presence of hydrocarbons under SLG^52^, etc. Figure 4 shows the detection of HT-2 with 4 SLG protected Cu samples which differ in geometry and provide resonances from 700 to 1000 nm. The amplitude sensitivity increases with resonance wavelength, while the phase sensitivity depends on the resonance darkness and varies from ~0.1 pg/mL to ~0.4 fg/mL. The sigmoidal fits for resonant wavelength (λ) and phase change (δΔ) as a function of concentration are shown by dotted lines in Fig. 4a,b. These yield a universal value of cooperativity *n*_*H*_ ~ 0.2 and variable *K*_*H*_. The negative cooperativity is most probably connected to the two dimensional nature of toxin-receptor interaction.

Non-specific binding on the surface of the sensor chip can also affect the results. A negative control study using neosolaniol^12^ was conducted on a SLG-protected Cu SPR biosensor (see Methods and Supplementary Information for details) to confirm selectivity. Each increase in neosolaniol concentration results in a resonant wavelength shift <1 nm, close to the limit of our ellipsometer’s spectral resolution. The very small shift of the resonant wavelength indicates non-specific binding does not affect the results. Our functionalization procedure and methods can be used for the detection of HT-2 in a commercial beer, as shown in Fig. S13.

## Conclusions

We have demonstrated a viable layered material platform for hybrid SPR biosensing. Graphene and hBN are excellent protectors of plasmonic properties or reactive metals with strong potential for biosensing functionalization. The life-time can be significantly enhanced by use of an additional thin layer of oxide deposited on the metal prior to SLG transfer or a transferred CNM encapsulating SLG. We realised extremely sensitive graphene protected copper SPR biosensors for HT-2 detection. We achieved a phase graphene protected copper SPR detection limit~0.5 fg/mL, 6 orders of magnitude lower than amplitude Au SPR. The layered material platform SPR biosensing could be used to further enhance specificity of molecular recognition elements. Our approach paves the way to realize novel biosensors with high sensitivity for point of care testing.

## Methods

### Film depositions

Cu is deposited on a cleaned glass substrate (size: 25 mm × 25 mm, thickness: 1 mm) by electron-beam evaporation at a base pressure ~10^−7^ mbar and growth rate ~0.3 nm/s. As an electron-beam target, we use 99.99% Cu from Sigma-Aldrich. A 1.5 nm Cr adhesion layer is evaporated onto the substrate before Cu.

### Metal protection by layered materials

#### SLG transfer

CVD SLG-on-Cu is covered by poly (methyl methacrylate) (PMMA) using spin coating. Then the PMMA film SLG attached is isolated by 25 g/L of ammonium persulfate solution that chemically etches Cu. The resulting PMMA-SLG is cleaned by deionized (DI) water and then transferred onto the target Cu. Next, the SLG covered Cu substrate is further annealed at 150 °C for 3 hours to enhance the adsorption. Finally, the PMMA layer is removed in acetone and the SLG surface left to dry in air for 6 hours.

#### h-BN transfer

CVD monolayer-h-BN is grown on Pt using ammonia borane as a precursor. The Pt foil is loaded into the center of a vacuum quartz tube placed in a furnace, and ammonia borane placed in a sub-chamber. The furnace is heated to 1100 °C under H_2_ gas (10 sccm). The sub-chamber is heated to 150 °C for the decomposition of ammonia borane. The growth of monolayer-h-BN on Pt is initiated by opening a valve of the sub-chamber. During growth for 30 min, the pressure is maintained at 0.13 Torr. After growth, the furnace is cooled down to room temperature under H_2_. Monolayer-h-BN is then transferred onto the target Cu substrate using electrochemical delamination method.

*Carbon Nanomembranes* are prepared from 4’-Nitro-1,1’-biphenyl-4-thiol (NBPT) (Taros, 95%, sublimated before use), as described in refs. ^23,53^. Electron beam irradiation is used to crosslink the molecules into a stable 1 nm film. Crosslinking is performed in high vacuum (<5 × 10^−8^ mbar) with an electron floodgun (Specs FG20) at 100 eV and a dose of 50 mC/cm^2^. The nitro group is reduced to an amino group, later used for bio-functionalization. CNMs are then transferred with a supporting PMMA film onto a SLG/Cu substrate. PMMA is then removed using acetone. The direct deposition of CNMs on a SPR chip is described in Supplementary Information.

### Graphene grafting

The protocol for graphene grafting with COOH terminal groups by electrochemical method comprises the following steps: First, a solution of 0.052 mmol of 4-NH_2_-3,5-F_2_PhCOOH with 60 mg of 85% H_3_PO_4_ and 25 ml of Milli-Q water. 12.8 mmol of imidazole is prepared. Second, an electrochemical cell is set up in a glass beaker using a Cu tape to fix the substrate, and to serve as electrode, a piece of Pt foil with surface area equal or larger than the conductive substrate area as the counter electrode, and a standard aqueous Ag/AgCl as reference electrode. All these electrodes are connected to a potentiostat. The chronoamperometry for the potentiostat is set to −0.4 V for 60 seconds. Third, 0.5 ml of a 0.1 M aqueous solution of NaNO_2_ are added to the previously prepared solution and shaken for 3 minutes. The freshly prepared solution is transferred to the cell (to cover the sample) and the electrochemical grafting is performed for~60 seconds. Finally, after disconnecting the electrodes, the substrate is washed with excess water and dried at room temperature under ambient conditions. If non-grafted by-products are present, an additional washing step is performed. E.g., for COOH containing impurities, the grafted sample is dipped into 1% NaOH, rinsed with water, then dipped into 1% acid (e.g. HCl or phosphoric), rinsed with an excess of water and dried.

### HT-2 biosensing protocol

To detect HT-2 selectively, a SLG-protected Cu SPR sensor chip needs to be functionalized by using 1-Pyrenebuturic acid N-hydroxy-succinimide ester as a linker and anti-HT-2 toxin Fab fragment as a receptor^12,13^. First, 1-Pyrenebuturic acid N-hydroxy-succinimide ester linker solution (2 mg/mL) in 100% MeOH is prepared. After sonication, the linker solution is incubated for 1 hour at room temperature, without shaking, to ensure solution saturation. Then we filter the saturated solution with a disposable filter unit attached to a syringe, and then put the sensor chip into the filtered solution. Filtering removes the undissolved linker and the resulting filtered solution is clear. After one-hour incubation, the chip is washed by pure 100% MeOH and 1 × PBS (pH 7.3). Then, the chip is transferred to 50 µg/ml of HT2-10 Fab solution in 1 × PBS (pH 5), and incubated for 20 min at room temperature. Next, the chip is moved from the antibody solution to 100 mM Ethanolamine solution (1 M Ethanolamine stock solution (pH 8.5) diluted 1:10 in distilled water), and incubated for 10 min. The Ethanolamine solution is used to block the linker without binding with receptor. Finally, the chip is washed with distilled water and stored in distilled water before SPR measurements.

For positive tests, 8 concentrations of HT-2 in 0.1 × PBS (pH 7.3) are prepared at 0.0001, 0.001, 0.01, 0.1, 1, 10, 100, and 1000 ng/ml. For negative tests, same concentrations of neosolaniol (1 mg/mL in 100% DMSO) in 0.1 × PBS (pH 7.3) are prepared.

### Ellipsometry measurements of SPR

The data are acquired using a focused beam M-2000F spectroscopic ellipsometer (J. A. Woollam, Inc.) with a beam size ~30 × 60 μm (for an angle of incidence of 59^0^). We record Ψ and Δ from 250 to 1700 nm with a 1 nm wavelength step. The amplitude ratio (tan Ψ) and phase difference (Δ) represent the change of outgoing polarization for *p-* and *s-*light so that $\tan (\Psi )exp(i\Delta )=\frac{{r}_{p}}{{r}_{s}}$, where *r*_*p*_ and *r*_*s*_ are the complex reflection coefficients for the *p-* and *s-*polarized light respectively^54^. The absence of air bubbles in the flow channel is checked with a CCD camera placed directly above the channel. The quality factor of a SPR curve is defined as the ratio of the resonance wavelength over the full width of the resonance at half maximum. Fresnel calculations for SLG protected Cu chips (used in bio-experiments) were performed in ref. ^11^. This showed a good agreement with measured data.

In the case of selective HT-2 detection, we deal with a surface chemistry described by the Hill equation which is non-linear in nature due to the dynamics of binding ligands to receptors. Hence, direct conversion of *r*_*p*_ to the refractive index changes is impossible in this case. Only for non-specific binding (with neosolaniol), as discussed in Supplementary Information, we observe a linear change of resonance wavelength shift with concentration.

## Supplementary information

Supplementary Information

## Data Availability

The authors declare that data supporting the findings of this study are available within the paper and its Supplementary Information Files. Additional data and codes are available from the corresponding author upon reasonable request.
