# Supplementary material for: Layered material platform for surface plasmon resonance biosensing
Source: Sci Rep. 2019 Dec 30;9:20286. doi: 10.1038/s41598-019-56105-7 (PMC6937298; doi:10.1038/s41598-019-56105-7)
Supplement: Supplementary file 1 — Supplementary Information [file 41598_2019_56105_MOESM1_ESM.pdf]

Supplementary information to:

"Layered materials platform for surface plasmon resonance biosensing"

F. Wu[1,2,10], P. A. Thomas[1], V. G. Kravets[1], H. O. Arola[3], M. Soikkeli[3], K. Iljin[3], G. Kim[4], M. Kim[5], H. S. Shin[4,5,6], D. V. Andreeva[7], C. Neumann[8], M. Küllmer[9], A. Turchanin[8], D. De Fazio[9], O. Balci[9], V. Babenko[9], B. Luo,[9] I. Goykhman[9], S. Hofmann[9], A. C. Ferrari[9], K.S. Novoselov[1,7,10], A. N. Grigorenko[1*]

[1]*School of Physics and Astronomy, University of Manchester, Manchester M13 9PL, UK*

[2] *Key Laboratory for Non-Equilibrium Synthesis and Modulation of Condensed Matter (Ministry of Education), School of Science, Xi'an Jiaotong University, Xi'an, Shaanxi 710049, China*

[3]*VTT Technical Research Centre of Finland Ltd., P.O. Box 1000, FI-02044 VTT, Espoo, Finland*

[4]*Department of Energy Engineering, Ulsan National Institute of Science & Technology (UNIST), Ulsan 44919, Republic of Korea*

[5]*Department of Chemistry, Ulsan National Institute of Science & Technology (UNIST), Ulsan 44919, Republic of Korea*

[6]*Low Dimensional Carbon Material Center, Ulsan National Institute of Science & Technology (UNIST), Ulsan 44919, Republic of Korea*

[7]*Department of Materials Science and Engineering, National University of Singapore, Singapore, 117575, Singapore*

[8]*Institute of Physical Chemistry, Friedrich Schiller University Jena, Lessingstraße 10, 07743 Jena, Germany*

[9]*Cambridge Graphene Centre, University of Cambridge, Cambridge CB3 OFA, UK*

[10]*Chongqing 2D Materials Institute, Liangjiang New Area, Chongqing, 400714, China*

I. Layered materials protection of metal SPR

1. Transfer protocols

Graphene

SLG can used to protect Cu SPR for long (~year) periods of time[1]. SLG is grown by chemical vapour deposition (CVD) on a 35µm-thick Cu foil, following the recipe in Ref. [2]. Cu is loaded in the growth chamber and the temperature (T) is raised up to 1000C in~200mTorr H_2. Then T is kept constant for 30mins. Next, 5 sccm of CH_4 are added to the 20 sccm H_2 flow to enable the growth process, which lasts 30mins. The sample is then cooled at~1 mTorr to room temperature (RT) and unloaded from the chamber.

The material is characterized by Raman spectroscopy using a Renishaw InVia spectrometer equipped with a 50X objective at 514nm. Figure S1 plots the Raman spectrum of SLG as-grown on Cu (red line), with Cu background photoluminescence removed[3]. The D peak is negligible at indicating negligible defects[4,5]. The 2D is a single Lorentzian, fingerprint of SLG[6].

SLG is then wet transferred on another 35µm-thick Cu, Fig. S2. A PMMA layer is spin-coated at the surface of SLG/Cu as a mechanical support and then it is dropped at the surface of a solution of ammonium persulfate (APS) and DI water for the etching of Cu. The PMMA membrane with attached SLG is then moved to a beaker filled with DI water for cleaning APS residuals. The PMMA+SLG membrane is lifted with the target Cu substrate. The material is once again characterized by Raman spectroscopy, Fig. S1, blue curve. The D peak is still absent, indicating that the transfer has not damaged the sample.

Fig. S3 plots the SPR resonances in Cu covered with SLG using the transfer method described above (along with an optical image). SPR curves measured ATR in air are shown

in Fig. S3b. They demonstrate deep surface plasmon resonances with almost zero reflection (Ψ_{min}~0.36°) at a resonance wavelength~605nm for a 45.6° incidence angle. These resonances are stable for at least one year in air.

hBN

hBN is a dielectric that has a large band gap[7] but is similar to SLG in crystalline lattice. As a result, hBN can protect metal SPR as efficiently as SLG. We use two transfer methods, P1 and P2.

P1: 1L-h-BN is grown by CVD on 100μm Fe foils (99.8%, Goodfellow) as described in Ref. [8]. Fe foils are cleaned in acetone and isopropanol and loaded into a custom cold-wall CVD system with a base pressure~10^{-6}mbar. T is ramped to 920°C as measured by an internal thermocouple in 1.5x10^{-2}mbar Ar (BOC, 99.9995%). The atmosphere is then switched to 10^{-2}mbar NH_3 (BOC, 99.98%) for 60mins. Borazine (Fluorochem) is added at a leak rate of 2x10^{-4}mbar for 2.5 hours to achieve full-coverage. The system is then cooled at a rate of 200°C/min. A scanning electron microscope (SEM) image of the as-grown film is in Fig. S4a. This consists of hBN domains with sizes ~100μm as seen from a shorter experiment with incomplete coverage in Fig. S4b.

hBN samples are then wet transferred. For this, PMMA is spin-coated on Fe/h-BN foils and the PMMA/h-BN is delaminated using electrochemical bubbling in a 1M-NaOH[8]. The PMMA/h-BN film is then transferred to sequential deionised water baths and lifted onto the target substrate. After drying and baking for 5 minutes at 120°C, the polymer is dissolved in acetone. To remove possible surface oxide that could appeared on the Cu substrates during air exposure or during transfer, the Cu/h-BN samples are annealed in 50 mbar Ar/H_2 (80%/20%) at 300°C for 1 hour.

P2: CVD 1L-hBN is grown on Pt foil using ammonia borane as a precursor[9]. The Pt foil is loaded into the centre of a vacuum quartz tube in a furnace, and ammonia borane is placed in a sub-chamber. The furnace is heated to 1100°C under 10sccm H_2. The sub-chamber is heated to 150 °C for the decomposition of ammonia borane. Growth is initiated by opening the sub-chamber valve. During growth, for 30min the pressure is maintained at 0.13 Torr. After growth, the furnace is cooled to RT under H_2. 1L-hBN is then transferred onto the target Cu substrate using electrochemical delamination.

Optical images of hBN on quartz/Cr(1.5nm)/Cu(43.5nm) fabricated by e-beam evaporation are shown in Fig. S5a. The surface of hBN-covered Cu is smooth with a small amount of visible defects. These do not influence the SPR resonance shown in Fig. S5b, with almost complete darkness (Ψ_{min}~0.8°) at 620nm for 45° incidence angle. However, the SPR properties, such as darkness of the resonance and the resonance quality factor, are not stable in air and water. Figure S5c shows the SPR deterioration (with increasing minimum reflection to Ψ_{min}~2°) measured in the same conditions as in Fig. S5b, after 13 days in air. At the same time, the SPR in water red shift (~2.5nm) with a characteristic time of 1 hour. The poor stability of the hBN protected samples is probably connected with defects.

Analogous results are obtained using P1. An optical image of the final structure is shown in Fig. S5d. Fig. S5e,f indicate excellent SPR amplitude and phase characteristics. These SPR curves survive for a period of months in air. However, the stability in water is not good. Figure S5g shows an image of the sample after measurements in ATR in water, indicating some surface deterioration. The evolution of SPR curves for hBN protected samples in water is shown in Fig. S5h. This confirms deterioration over ~1 day.

Carbon nanomembranes

1nm thick CNMs prepared via low energy e-beam induced crosslinking of self-assembled monolayers (SAMs) of 4’-nitro-1,1’-biphenyl-4-thiol (NBPT)[10] are transferred onto SLG covering Cu using a sacrificial PMMA film. The transfer is characterised using a stereomicroscope (Zeiss) enabling imaging of the whole sample. Bright field (Fig. S6a) and dark field (Fig. S6b) images confirm transfer without macroscopic defects. Figs. S6c,d are optical images of SLG protected Cu before and after CNM transfer. Comparing a smooth and flat SLG protected Cu, Fig. S6c, with the final structure, Fig. S6d, we observe that CNM transfer introduces some additional roughness. Figures S6e shows the initial excellent SPR of SLG protected Cu at 570 nm and 49.7° incidence. CNM adds a dielectric layer to the structure, which changes the angle at which the resonance is observed to 52.7°. The SPR remains completely dark at the resonance minimum, with a small red shift (~ 2 nm) of the resonant wavelength caused by CNM, Fig. S6f. The SPR keeps high quality and darkness even after CNM transfer and functionalization. The quality is maintained for at least 8 months, Fig. S6g, while the position of the SPR dip at~57.9° shifts to red (~ 14 nm of shift) after 14 months, Fig. S6h.

2. Direct growth

Graphene and hBN

Figure S7a shows an optical image of a sample where SLG is directly grown 43.5nm Cu by CVD. This significantly increases the Cu roughness. As a result, the SPR curve becomes broad, Fig. S7b, and Ψ_{min} in the air cannot reach zero, which makes ultra-sensitive biosensing difficult[11,12]. The increased roughness can be due to the high temperatures arising during CVD.

CVD hBN on a SPR film also induces deterioration of the metal surface to such degree that the SPR resonances are of extremely poor quality (not shown here).

CNMs

Cu/glass substrates are introduced in a UHV Multiprobe system (Scienta Omicron) with a base pressure < 2x 10^{-10} mbar. The Cu substrates are in situ cleaned by Ar^+ sputtering (1 keV, 10 mA, Focus) at~2x10^{-7} mbar for 10 minutes until no carbon is detected by X-ray photoelectron spectroscopy (XPS, Scienta Omicron, monochromatized Al Kα X-ray source with Argus energy analyser that has a resolution of 0.6 eV). The NBPT SAMs are prepared via vapour deposition for 30 minutes using a Knudsen-type evaporator (TCE-BSC, Kentax) from a quartz crucible heated to 100 °C. Heating of NBPT increases the chamber pressure to~10^{-6} mbar, as detected by N_2-calibrated vacuum ion gauge. The Cu substrates are kept at RT during vapour deposition. Figure S8a presents high resolution XP spectra (C1s, N1s and S2p) confirming the SAM growth on the Cu/glass substrate. The molecules are covalently bound to Cu by thiolate, as confirmed by the sulphur signal, and form a 1.2 nm film, as calculated from the attenuation of the Cu2p signal before and after growth. The N1s signal consist of two species, attributed to the nitro group of the NBPT molecules at a binding energy (BE)~405.7eV[13] and an amino group (BE 398.8 eV) arising from partially reduced molecules[13]. Afterwards SAMs are crosslinked into CNMs in the same UHV chamber using a low energy electron gun (NEK-150, STAIB Instruments) at 50eV and an electron dose~50 mC/cm^2 and monitored again by XPS. Due to the irradiation, the C1s signal is broadened as new chemical species are created by crosslinking and the thickness is slightly decreased due to desorption of detached fragments. Furthermore, the nitro signal is completely reduced to amino groups and the sulphur signal is modified by the creation of copper sulphides. These results are in good agreement with previously published CNMs grown via vapour deposition in UHV[14].

An optical image of a sample is shown in Fig. S8b. The direct growth of CNM films suppresses plasmonic properties of the final structure. Indeed, the SPR curves measured in air

for a directly grown sample, Fig. S8c, are not as good as the SPR curves of the samples obtained by CNM transfer, Fig. S6, with minimum Ψ_{min}~5° (which corresponds to the minimum reflection~4%) at 45° incidence. After keeping the sample in air for 6 months, the SPR wavelength redshifts ~26 nm and the corresponding incident angle is~47.9°, Fig. S8d. Thus, some Cu oxidation (or relaxation of the structure) still happens even after the growth of CNM. Fig S8e plots the SPR response in water. The SPR resonance wavelength shifts significantly over 2 days. Thus, a CNM layer grown on a metal substrate cannot be used for effective protection of metals for biosensing. Most likely, roughness is introduced due to the sputtering of the sample before SAM growth and the crosslinking with low energy electrons into CNMs. This might be overcome by optimisation of the fabrication parameters.

3. Metal oxide

To increase protection, an additional layer of oxide can be added. Even a thin (~10nm) metal oxide layer evaporated on Cu can protect the SPR properties. Fig.S9a shows the SPR curves of 10nm HfO_2 covered Cu. We get excellent SPR curves with narrow (quality factor ~10) and dark (reflection at the resonance minimum <0.1%) resonances. At the same time, the oxide thickness can be used to tune the resonant wavelength, Fig. S9a.

Metal oxide also works well for Ag SPR protection, Fig. S9b. Al_2O_3 and HfO_2 films are evaporated on the Ag films. Excellent Ag SPR curves are observed in all cases, Fig. S9b. The sensitivity of HfO_2 (8 nm) / Al_2O_3 (8 nm) covered Ag on glycerol-water was checked, as for Figs. S9c,e. Figs. S9d,f plot the resonant wavelength dependence on the refractive index of a water-glycerol mixture. The corresponding sensitivity is ~10^3 and 10^4 nm/RIU.

Since it is more difficult to arrange bio-functionalization for a dielectric material, these additional oxide layers can be covered with SLG to make use of SLG functionalization as described below.

II. Functionalization for biosensing

Functionalization of SLG-covered Cu SPR sensors is necessary to achieve selectivity and to detect the target molecules (SPR respond to the overall change of local refractive index[15]). The electrochemical protocol of functionalization with COOH is as follows:

1) Place 0.052 mmol 4-NH_2-3,5-F_2PhCOOH into a glass vial.

2) Add 60 mg of 85% H_3PO_4.

3) Add 25 ml of Milli-Q water and make sure that all solid is dissolved.

4) Add 12.8 mmol of imidazole. Close the lid and shake until imidazole dissolves completely.

5) Prepare the electrochemical cell. Take a 25 ml beaker and, using Cu tape, fix the sample-working electrode, a Pt foil counter electrode, with surface ≥ than the substrate conductive area, and position 1-5 mm away a standard aqueous Ag/AgCl reference electrode. All should be connected with corresponding clips to a potentiostat.

6) Perform chronoamperometry at -0.4V for 60 s.

7) Add 0.5 ml of 0.1M aqueous solution of $NaNO_2$ and shake for 3 minutes.

8) Transfer the contents into the cell (just enough to cover the sample).

9) Start electrochemical grafting (60 seconds).

10) Once finished, disconnect electrodes, take out the sample and wash it with excess water. Dry naturally in the air. In case of COOH containing impurities: dip the grafted sample into 1% NaOH, then rinse with water, then dip into 1% acid (e.g. HCl or phosphoric), rinse with excess of water, dry naturally.

Figure S10 shows functionalization of COOH on SLG-protected Cu. Figures S10a,c show optical images before and after functionalization. Figures S10b,d plot the corresponding angle-dependent SPR curves in air. For SLG-covered Cu, the SPR dip occurs at 45.2°, λ~591

nm and $\Psi_{min} \sim 0.1°$. After COOH functionalization, at 45° the dip of curve increases ($\Psi_{min} \sim 3°$) and the resonance becomes broader, which can be caused by COOH group bonded to the SLG surface, resulting in refractive index variation around the Cu surface, thus changing the incident angle for the SPR dip.

An analogous protocol can be used to functionalize SLG with NH_2 groups.

III. Biosensing of HT-2

1. Graphene protected copper

The functionalization of SLG protected Cu can be done using biochemistry. To detect HT-2, the SPR sensor needs to be functionalized using 1-Pyrenebuturic acid N-hydroxy-succinimide ester as linker and anti-HT-2 toxin Fab fragments as a receptor[16]. First, 1-Pyrenebuturic acid N-hydroxy-succinimide ester linker solution (2 mg/mL) in 100 % MeOH is prepared. After sonication, the linker solution is incubated for 1 h at RT without shaking to ensure saturation. We then filter the saturated solution with a disposable filter unit attached to a syringe, and then put the sensor chip into the filtered solution. Filtering removes the undissolved linker and the resulting solution is clear. After one-hour incubation, the chip is washed by pure 100 % MeOH and 1 × PBS (pH 7.3). Then, it is transferred to 50 µg/ml of HT2-10 Fab solution in 1 × PBS (pH 5), and incubated for 20 min at room temperature. Next, the chip is moved from the antibody solution to 100 mM Ethanolamine (1M Ethanolamine stock solution (pH 8.5) diluted 1:10 in distilled water), and incubated for 10 mins. The Ethanolamine solution is used to block the unreacted active sites in the linker. Finally, the chip is washed with distilled water and stored in distilled water before SPR measurements.

The SPR response of SLG-protected Cu for HT-2 detection is checked in air, Fig. S11a. The SPR dip occurs at $\theta = 45°$, $\lambda_R = 597$ nm and $\Psi_{min} \sim 0^o$. After functionalization with the linker (1-Pyrenebuturic acid N-hydroxy-succinimide ester) and receptor (HT2-10 Fab

fragment), we check the SPR curve in PBS solution (Fig. S11b), and find that at $\theta = 59°$ the SPR dip increases ($\Psi_{min} \sim 4.6°$ at $\lambda_R = 734$ nm) and the resonance becomes broader. Then SPR ellipsometric spectra are measured in PBS buffer solution after pumping different concentrations of HT-2 (0.0001, 0.001, 0.01, 0.1, 1, 10, 100, 1000ng/ml) into the flow cell. Figure S11c shows the change of Ψ with wavelength at 59° after functionalization of 1000 ng/ml HT-2. The value where Ψ_{min} reaches zero can be attributed to the binding of HT-2 with HT2-10 Fab.

2. Biacore T200 benchmarking

We perform benchmarking experiments of HT-2 detection with HT2-10 Fab fragments using a commercial Biacore T200 instrument (GE Healthcare) and Sensor Chip CM5 with carboxymethylated dextran matrix (GE Healthcare) at 25 °C. We use EDC/NHS chemistry to immobilize HT2-10 Fab fragments on an the active surface and anti-mycophenolic acid (MPA) Fab fragment, which does not cross-react with HT-2, on the reference surface. 1 x PBS-P (20 mM phosphate buffer, 2.7 mM KCl, 0.137 M NaCl, 0.05% P20, GE Healthcare) supplemented with 0.1% DMSO is used as running buffer. Six concentrations of HT-2 (0, 0.1, 1, 10, 100 and 1000 ng/ml) in 1 x PBS-P with 0.1% DMSO are injected at a flow rate of 30 µl/min for 3 mins. After dissociation for 6 mins, regeneration is done with 10 mM glycine-HCl, pH 2 (contact time 30 s, flow rate: 30 µl/min) followed by stabilization for 90s. All samples are analyzed in replicates. The results are analyzed with the Evaluation T200 - software with a 1:1 Langmuir binding model.

The obtained sensorgrams are shown in Fig. S12. This yields an amplitude detection limit~1 ng/ml with the Biacore T200. This is 6 orders of magnitude worse than the phase detection limit of our SLG-protected Cu chips. The extracted constants from a Langmuir model are: the absorption rate ka~10^6 1/(Ms), kd~0.03 1/s and KD~10^{-8} M.

3. Detection of HT-2 toxin in canned beer.

To check that SLG-protected Cu chips can be applied to detection of HT-2 toxin in a commercial beer, we make use of GPC SPR chips functionalized by HT2-10 Fab fragments as described above. Then, we measure the SPR curves in PBS buffer solution before and after pumping a popular canned beer through the flow cell containing SPR chip. Figure S13 shows that the shift of resonance is negligible (<0.1nm) in both amplitude and phase spectra. The change of the phase is <0.8° which, combined with a sensitivity of 9°/(pg/mL), gives a HT-2 toxin concentration <100 fg/mL. This is well below 10 ng/mL, which provides a tolerable daily intake for a 70 kg person drinking one pint of beer a day[17].

4. Negative control of graphene-protected copper SPR biosensor using neosolaniol

Non-specific binding on the surface of sensor chip can affect the results of biosensing. In addition, the change of volume concentration of some substance in a solution can change the refractive index of a solution and hence affect the results of biosensing. A negative control study using neosolaniol is thus conducted on our SLG protected Cu SPR biosensors (see Methods for details). The inset in Fig. S14b provides the chemical structure of neosolaniol. Figure S14a shows the change of SPR spectral dependence for a SLG protected Cu SPR biosensor subject to different concentrations of neosolaniol. The corresponding change of the resonance position (resonant shift) is shown in Fig. S14b. The resonance spectral shift is 3 times smaller than the corresponding shits for a sensor subject to HT-2. The changes of resonance wavelength in case of negative control is linear as a function of concentration, in contrast to the change associated with specific (surface) binding, which follows the Hills law. Hence, nonspecific binding can take place, but does not affect our results.

Supplementary References

1 Kravets, V. G. *et al.* Graphene-protected copper and silver plasmonics. *Scientific Reports* **4**, 5517, doi:10.1038/srep05517 (2014).

2 Li, X. *et al.* Large-Area Synthesis of High-Quality and Uniform Graphene Films on Copper Foils. *Science* **324**, 1312, doi:10.1126/science.1171245 (2009).

3 Lagatsky, A. A. *et al.* 2 μm solid-state laser mode-locked by single-layer graphene. *Applied Physics Letters* **102**, 013113, doi:10.1063/1.4773990 (2013).

4 Ferrari, A. C. & Basko, D. M. Raman spectroscopy as a versatile tool for studying the properties of graphene. *Nature Nanotechnology* **8**, 235, doi:10.1038/nnano.2013.46 (2013).

5 Cançado, L. G. *et al.* Quantifying Defects in Graphene via Raman Spectroscopy at Different Excitation Energies. *Nano Letters* **11**, 3190-3196, doi:10.1021/nl201432g (2011).

6 Ferrari, A. C. *et al.* Raman Spectrum of Graphene and Graphene Layers. *Physical Review Letters* **97**, 187401, doi:10.1103/PhysRevLett.97.187401 (2006).

7 Kim, G. *et al.* Catalytic Conversion of Hexagonal Boron Nitride to Graphene for In-Plane Heterostructures. *Nano Letters* **15**, 4769-4775, doi:10.1021/acs.nanolett.5b01704 (2015).

8 Caneva, S. *et al.* Controlling Catalyst Bulk Reservoir Effects for Monolayer Hexagonal Boron Nitride CVD. *Nano Letters* **16**, 1250-1261, doi:10.1021/acs.nanolett.5b04586 (2016).

9 Kim, G. *et al.* Growth of High-Crystalline, Single-Layer Hexagonal Boron Nitride on Recyclable Platinum Foil. *Nano Letters* **13**, 1834-1839, doi:10.1021/nl400559s (2013).

10 Turchanin, A. & Gölzhäuser, A. Carbon Nanomembranes. *Advanced Materials* **28**, 6075-6103, doi:10.1002/adma.201506058 (2016).

11 Malassis, L. *et al.* Topological Darkness in Self-Assembled Plasmonic Metamaterials. *Advanced Materials* **26**, 324-330, doi:10.1002/adma.201303426 (2013).

12 Kravets, V. G. *et al.* Singular phase nano-optics in plasmonic metamaterials for label-free single-molecule detection. *Nature Materials* **12**, 304, doi:10.1038/nmat3537 (2013).

13 Kankate, L., Turchanin, A. & Gölzhäuser, A. On the Release of Hydrogen from the S−H groups in the Formation of Self-Assembled Monolayers of Thiols. *Langmuir* **25**, 10435-10438, doi:10.1021/la902168u (2009).

14 Matei, D. G. *et al.* Functional Single-Layer Graphene Sheets from Aromatic Monolayers. *Advanced Materials* **25**, 4146-4151, doi:10.1002/adma.201300651 (2013).

15 Homola, J., Yee, S. S. & Gauglitz, G. Surface plasmon resonance sensors: review. *Sensors and Actuators B: Chemical* **54**, 3-15, doi:10.1016/S0925-4005(98)00321-9 (1999).

16 Arola, H. O. *et al.* Specific Noncompetitive Immunoassay for HT-2 Mycotoxin Detection. *Analytical Chemistry* **88**, 2446-2452, doi:10.1021/acs.analchem.5b04591 (2016).

17 Peters, J. *et al.* Mycotoxin profiling of 1000 beer samples with a special focus on craft beer. *PloS one* **12**, e0185887-e0185887, doi:10.1371/journal.pone.0185887 (2017).

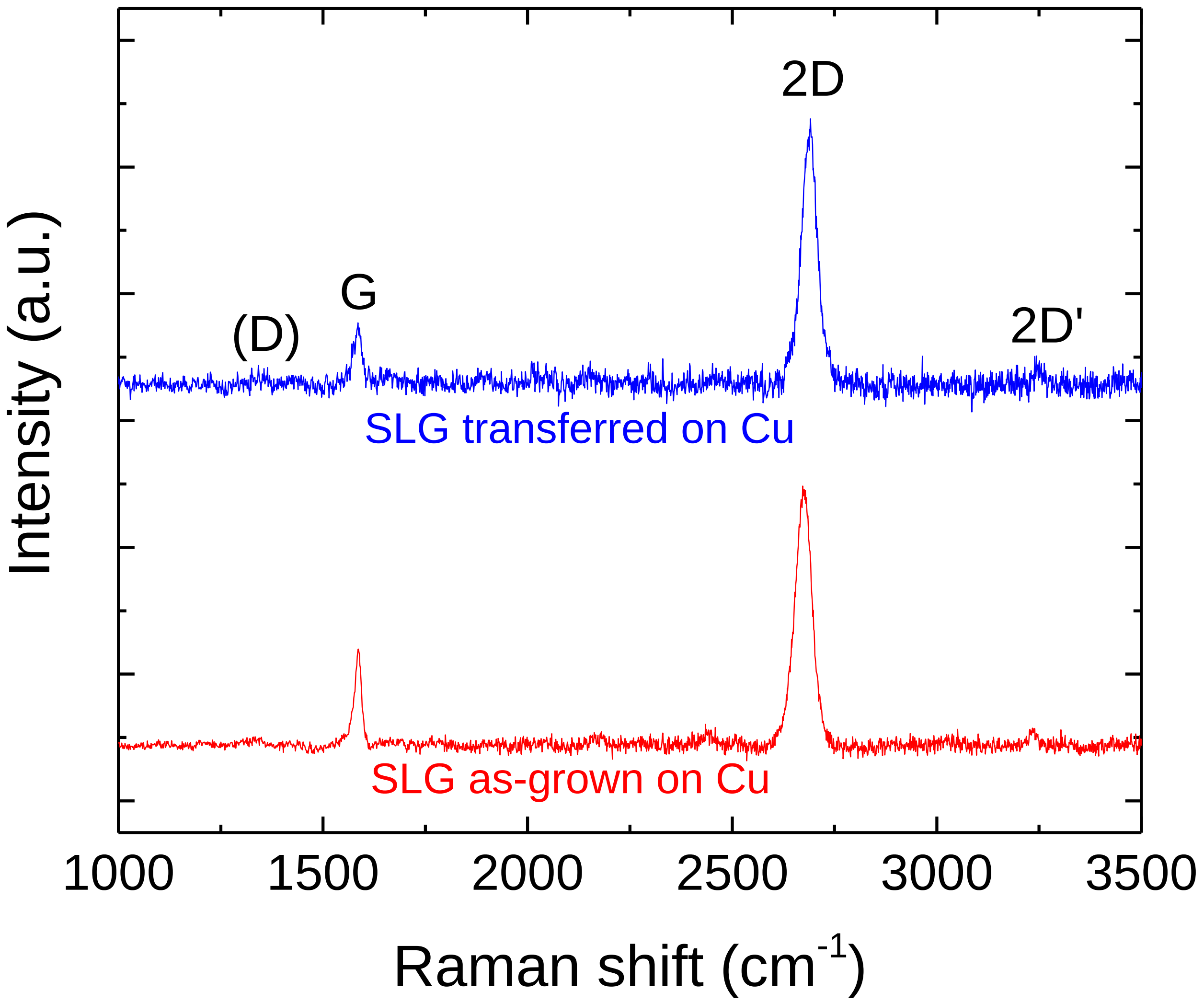

Figure S1 | Representative Raman spectra at 514nm of SLG as-grown (the red curve) and transferred on Cu SPR chip (the blue curve).

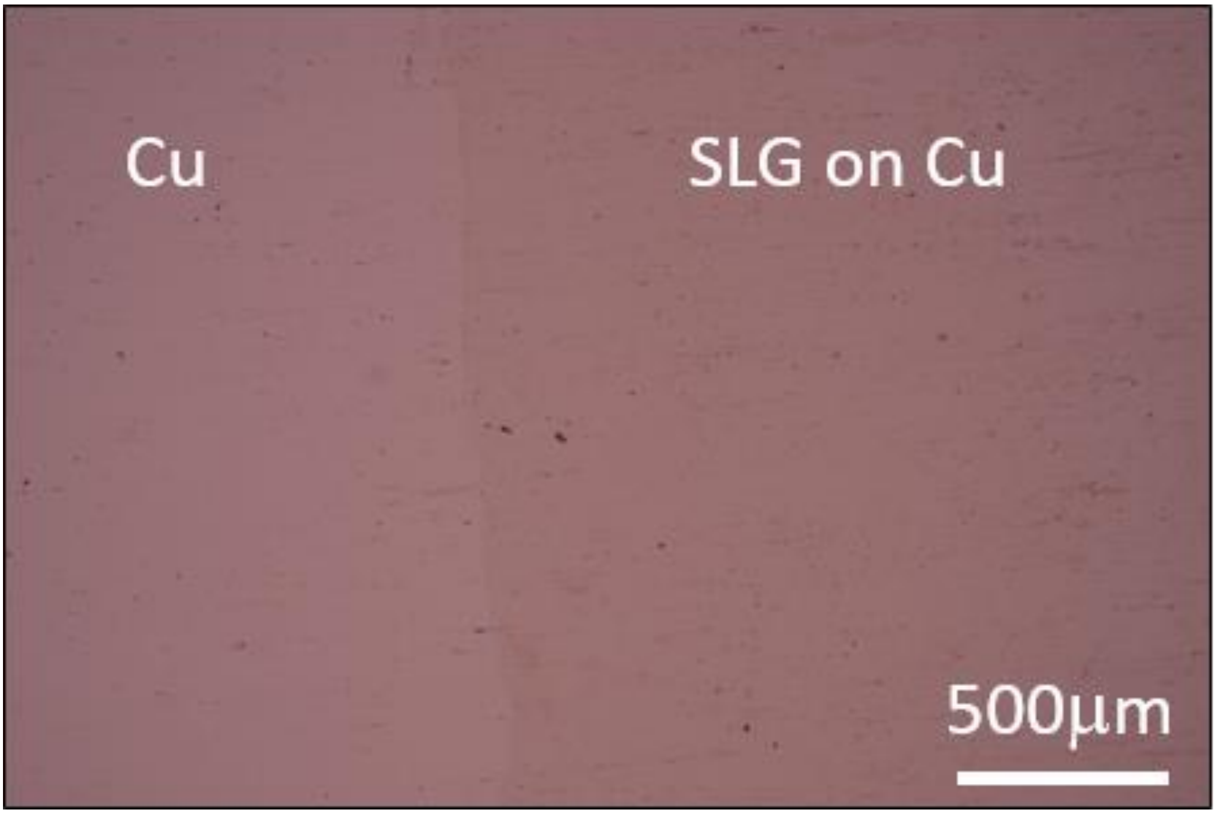

Figure S2 | Optical image of SLG transferred on Cu, at the edge of SLG.

a b

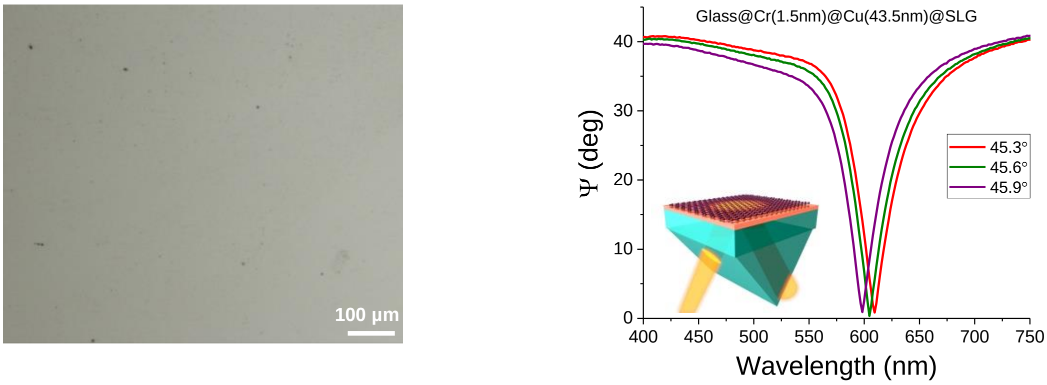

Figure S3 | SLG-protected Cu plasmonics. a, Optical image of a SLG-protected Cu from P1. **b,** Angle-dependent SPR response of the sample shown in **a**. The data are measured with ATR geometry in air.

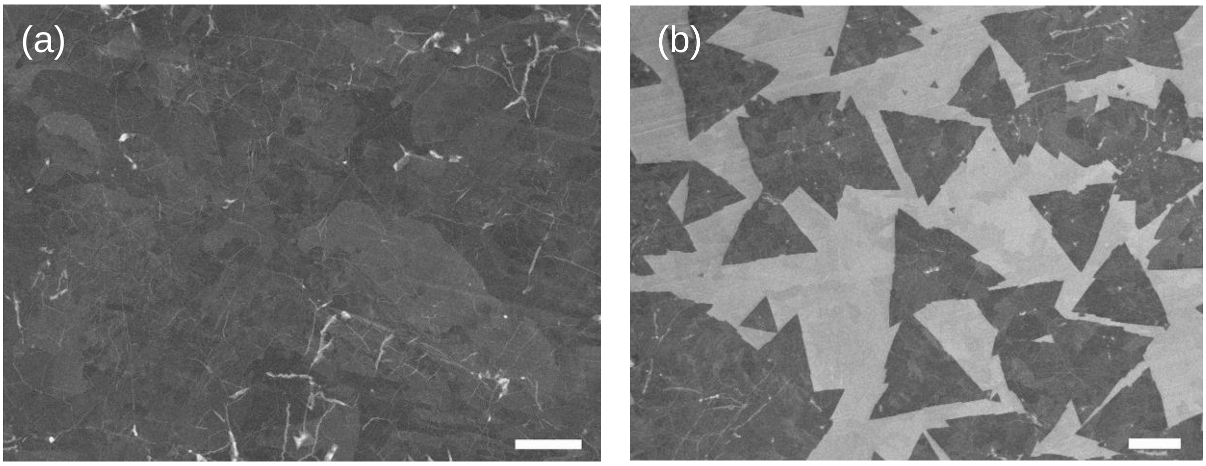

Figure S4 | hBN growth. SEM image of (a) full-coverage hBN film; (b) SEM partial coverage sample showing domain sizes before they merge to form a film. Scale bar: (a) 50μm; (b) 100μm.

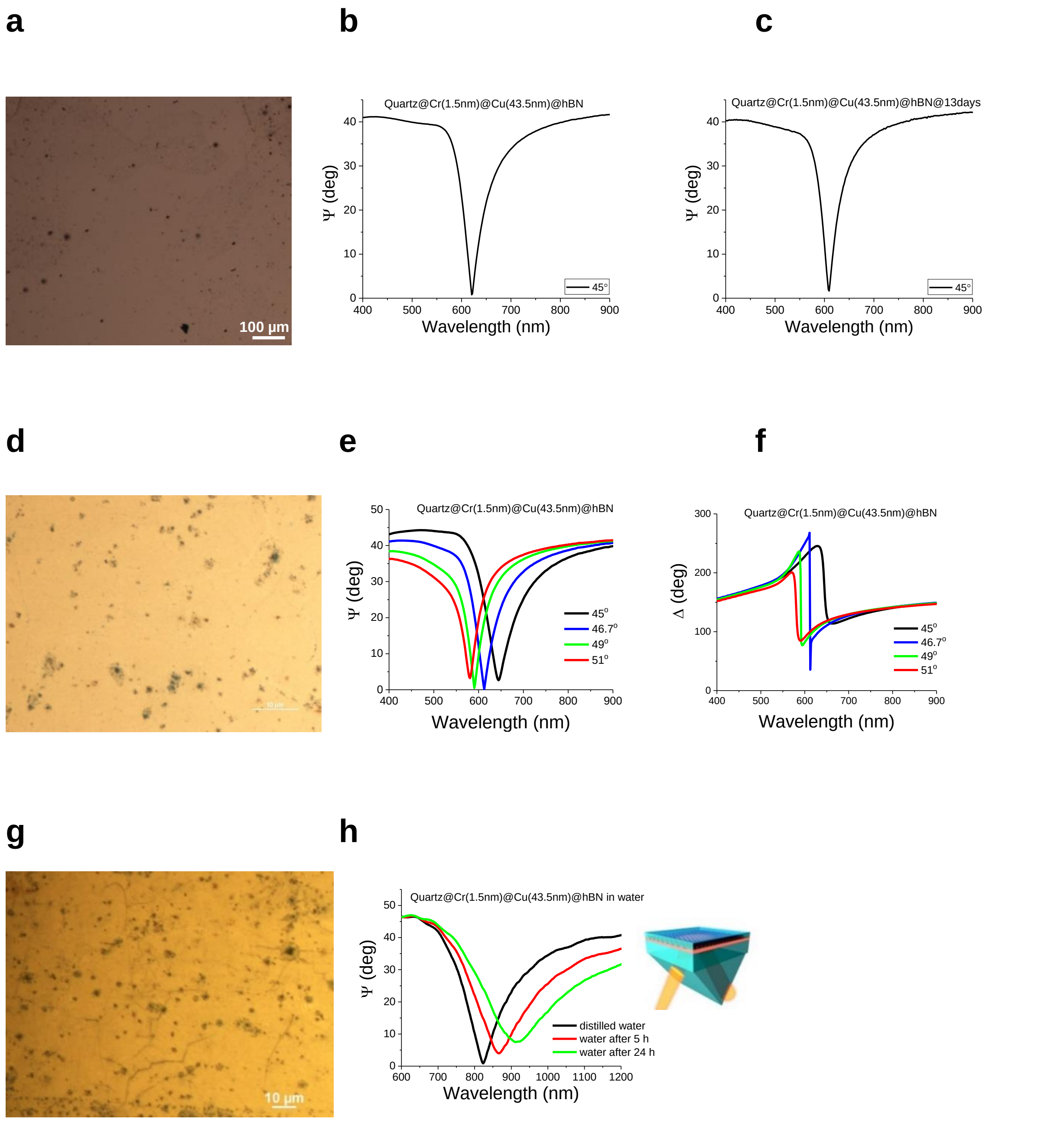

Figure S5 | hBN protected Cu plasmonics. a, Optical image of hBN protected Cu from P2. **b-c,** SPR response of as-prepared sample and after 13 days in air. The incident angle is 45°. **d,** Optical image of hBN protected Cu form P1. **e-f,** SPR response, amplitude Ψ and phase Δ. **g,** Optical image after SPR measurements in water. **h,** SPR curves measured in ATR in water.

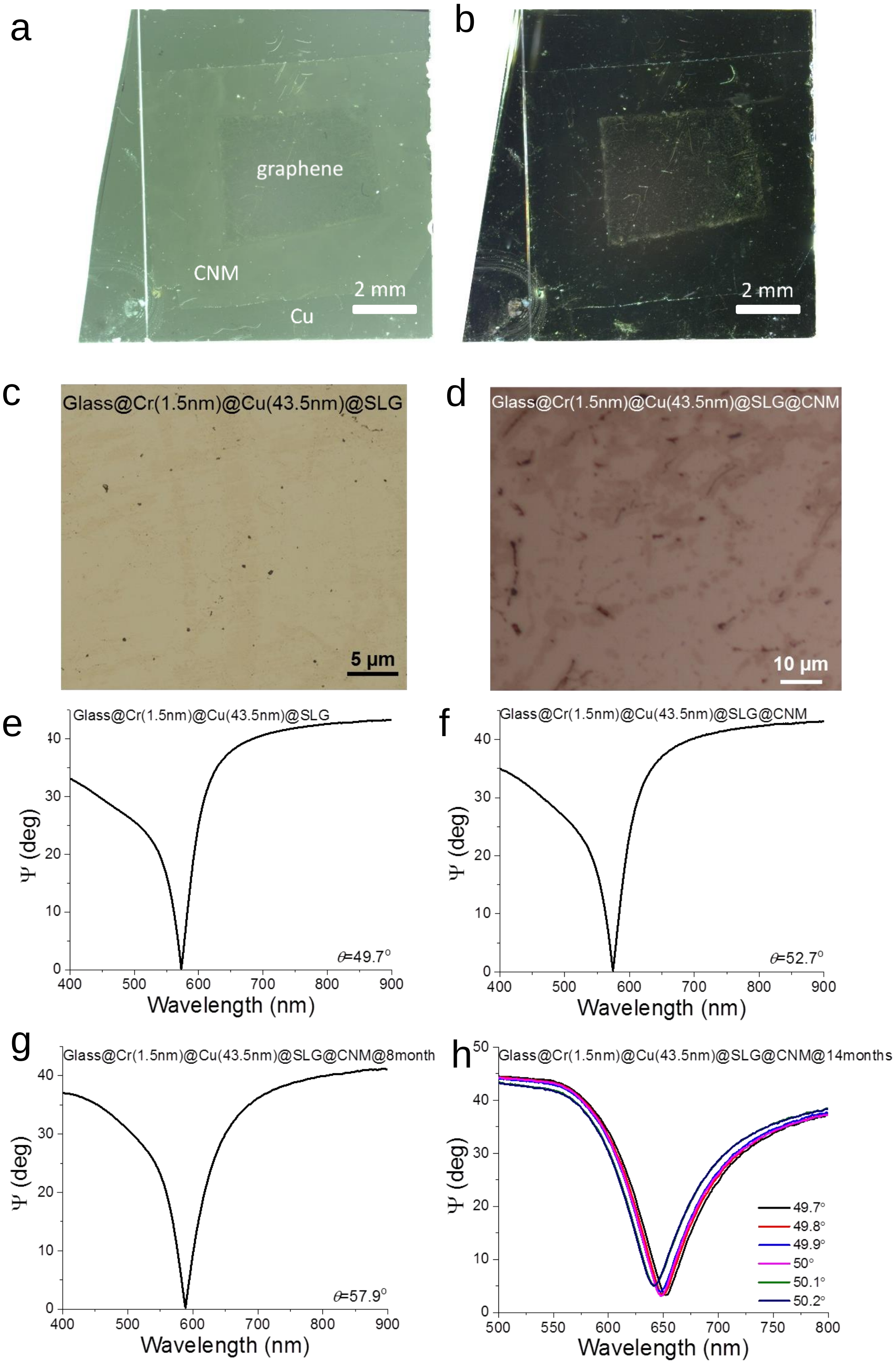
a
graphene
CNM
Cu
2 mm
b
2 mm
c
Glass@Cr(1.5nm)@Cu(43.5nm)@SLG
5 μm
d
Glass@Cr(1.5nm)@Cu(43.5nm)@SLG@CNM
10 μm
e
Glass@Cr(1.5nm)@Cu(43.5nm)@SLG
Ψ (deg)
θ=49.7°
Wavelength (nm)
f
Glass@Cr(1.5nm)@Cu(43.5nm)@SLG@CNM
Ψ (deg)
θ=52.7°
Wavelength (nm)
g
Glass@Cr(1.5nm)@Cu(43.5nm)@SLG@CNM@8month
Ψ (deg)
θ=57.9°
Wavelength (nm)
h
Glass@Cr(1.5nm)@Cu(43.5nm)@SLG@CNM@14months
Ψ (deg)
49.7°
49.8°
49.9°
50°
50.1°
50.2°
Wavelength (nm)

Figure S6 | CNM transfer on SLG-protected Cu. a-b, Optical bright field and dark field images of SLG-protected Cu after CNM transfer. **c-d,** Optical image of SLG-protected Cu before and after CNM transfer. **e-f** SPR response in air. **g,** Ψchange for SLG-covered Cu with CNM after 8 months air exposure. **h,** Angle-dependent SPR change in air after 14 months.

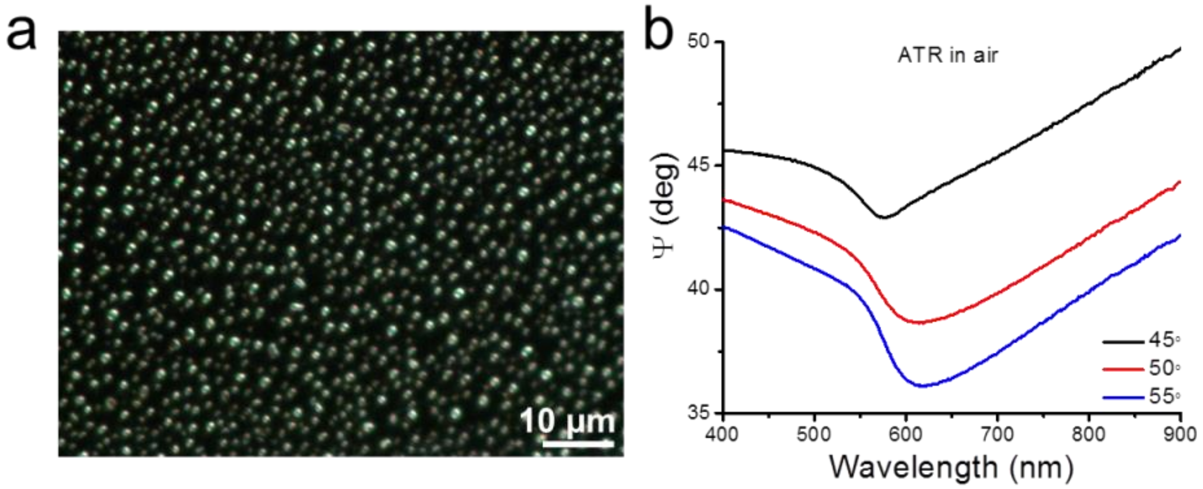

Figure S7 | SLG growth on Cu. a, Optical image of CVD SLG grown on Cu. **b,** Angle-dependent SPR response in air.

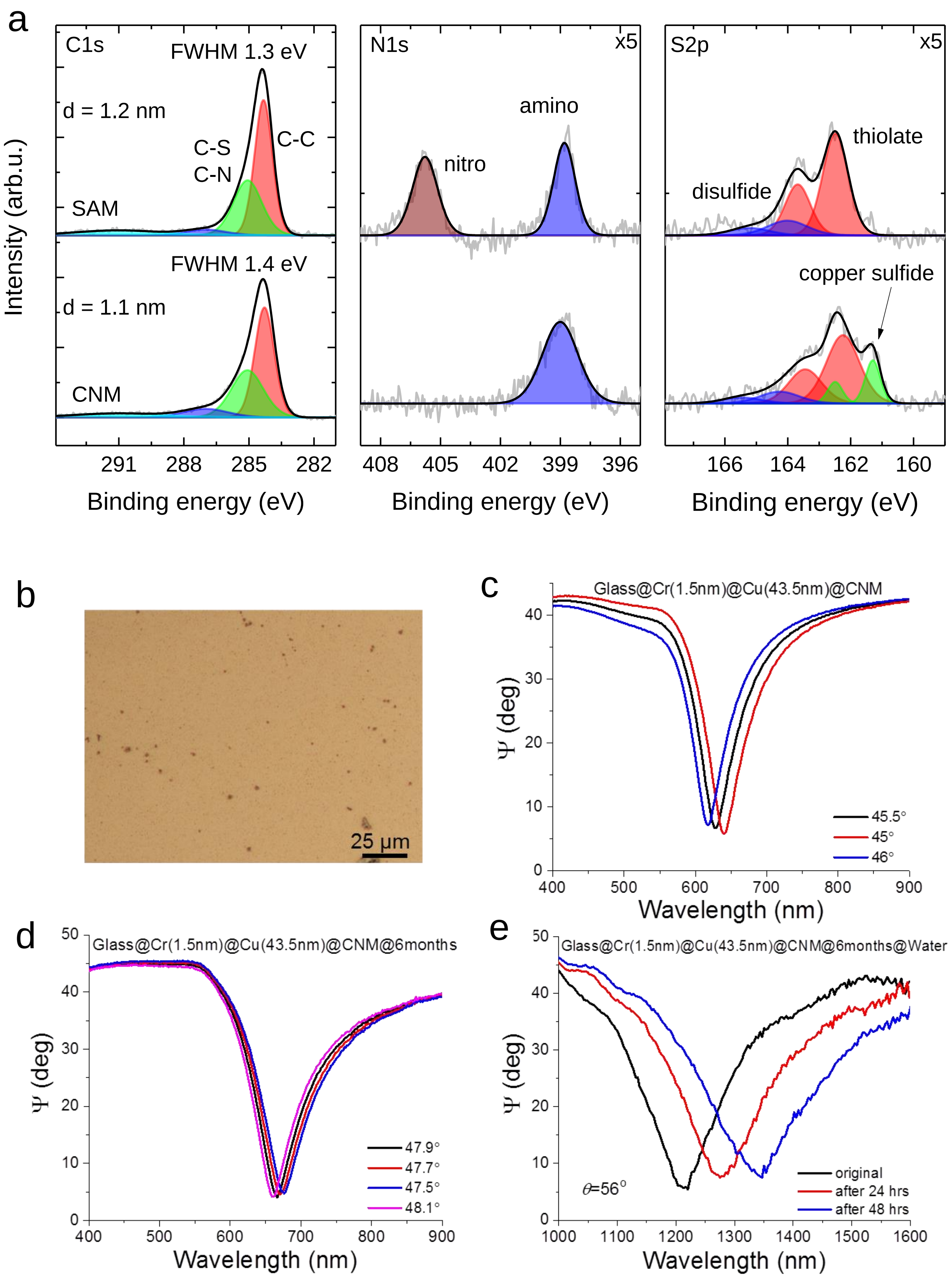
a
C1s
FWHM 1.3 eV
d = 1.2 nm
C-S
C-N
C-C
SAM
FWHM 1.4 eV
d = 1.1 nm
CNM
Intensity (arb.u.)
291 288 285 282
Binding energy (eV)
N1s
x5
amino
nitro
408 405 402 399 396
Binding energy (eV)
S2p
x5
thiolate
disulfide
copper sulfide
166 164 162 160
Binding energy (eV)
b
25 µm
c
Glass@Cr(1.5nm)@Cu(43.5nm)@CNM
Ψ (deg)
45.5°
45°
46°
Wavelength (nm)
d
Glass@Cr(1.5nm)@Cu(43.5nm)@CNM@6months
Ψ (deg)
47.9°
47.7°
47.5°
48.1°
Wavelength (nm)
e
Glass@Cr(1.5nm)@Cu(43.5nm)@CNM@6months@Water
Ψ (deg)
θ=56°
original
after 24 hrs
after 48 hrs
Wavelength (nm)

Figure S8 | CNM growth on Cu. a, XP spectra (C1s, N1s and S2p) after SAM growth and e-induced irradiation into a CNM. The intensity of the N1s and S2p spectra is multiplied by 5. **b,** Optical image of CNM grown on Cu. **c-d,** Ψ change for as-prepared samples and after air exposure for 6 months. **e,** Stability in water.

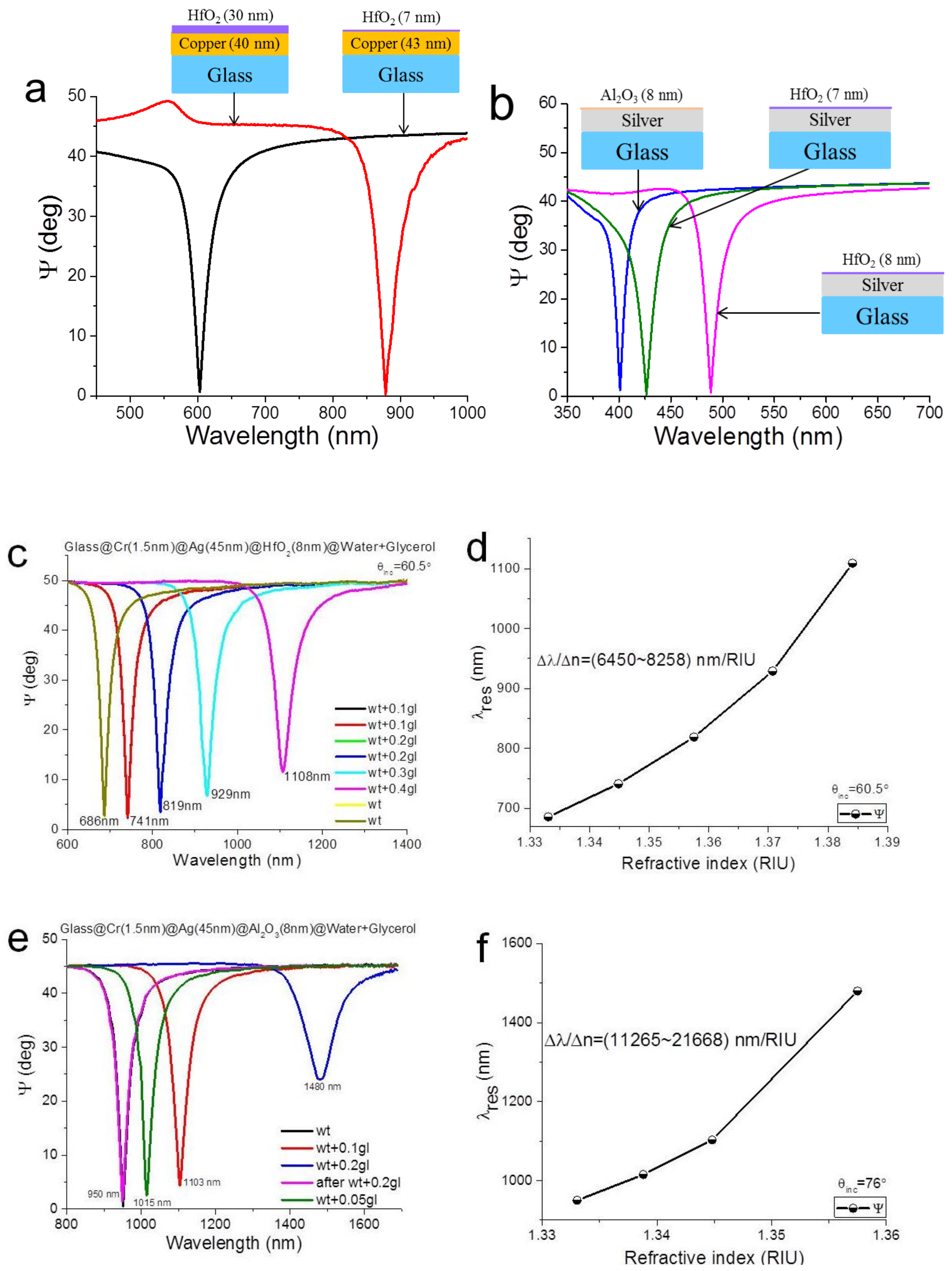

Figure S9 | Metal oxide for Cu and Ag protection. a, SPR curves for different thickness of HfO_2 protected Cu. **b,** SPR response of HfO_2/Al_2O_3 protected Ag. **c,** Ψ change for 8nm HfO_2

covered Ag for different concentrations of glycerol-water. **d,** λ_{res} dependence on refractive index in water-glycerol. **e,** SPR curves of 8nm Al_2O_3 covered Ag for different concentrations of glycerol-water. **f,** Corresponding λ_{res} dependence on refractive index of water-glycerol.

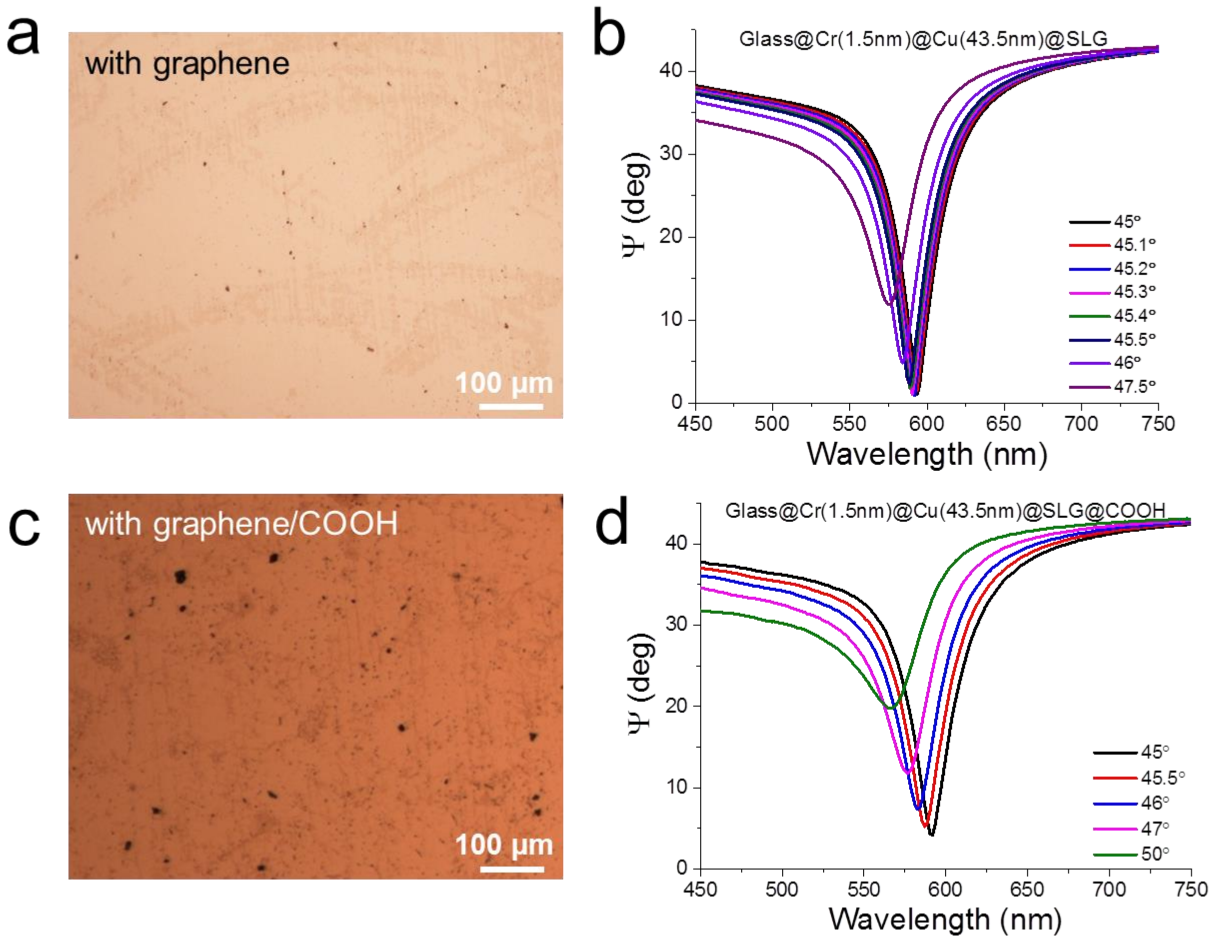

Figure S10 | SLG functionalized with COOH. a, Optical image of SLG-protected Au. **b,** Corresponding SPR response in air. **c,** Optical image after SLG functionalization with COOH. **d,** Corresponding SPR response after SLG functionalization checked by ATR in air.

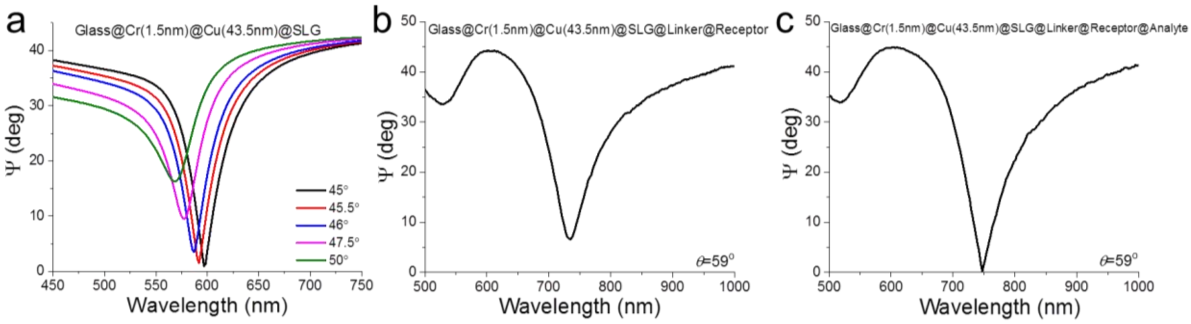

Figure S11 | SLG functionalization for HT-2 detection. a, Angle-dependent SPR response of SLG-protected Cu selected for further functionalization. **b,** Ψ change after SLG functionalization with linker and receptor. The incident angle is 59°. **c,** SPR of the same sample after reacting with 1000 ng/ml of HT-2.

a

b

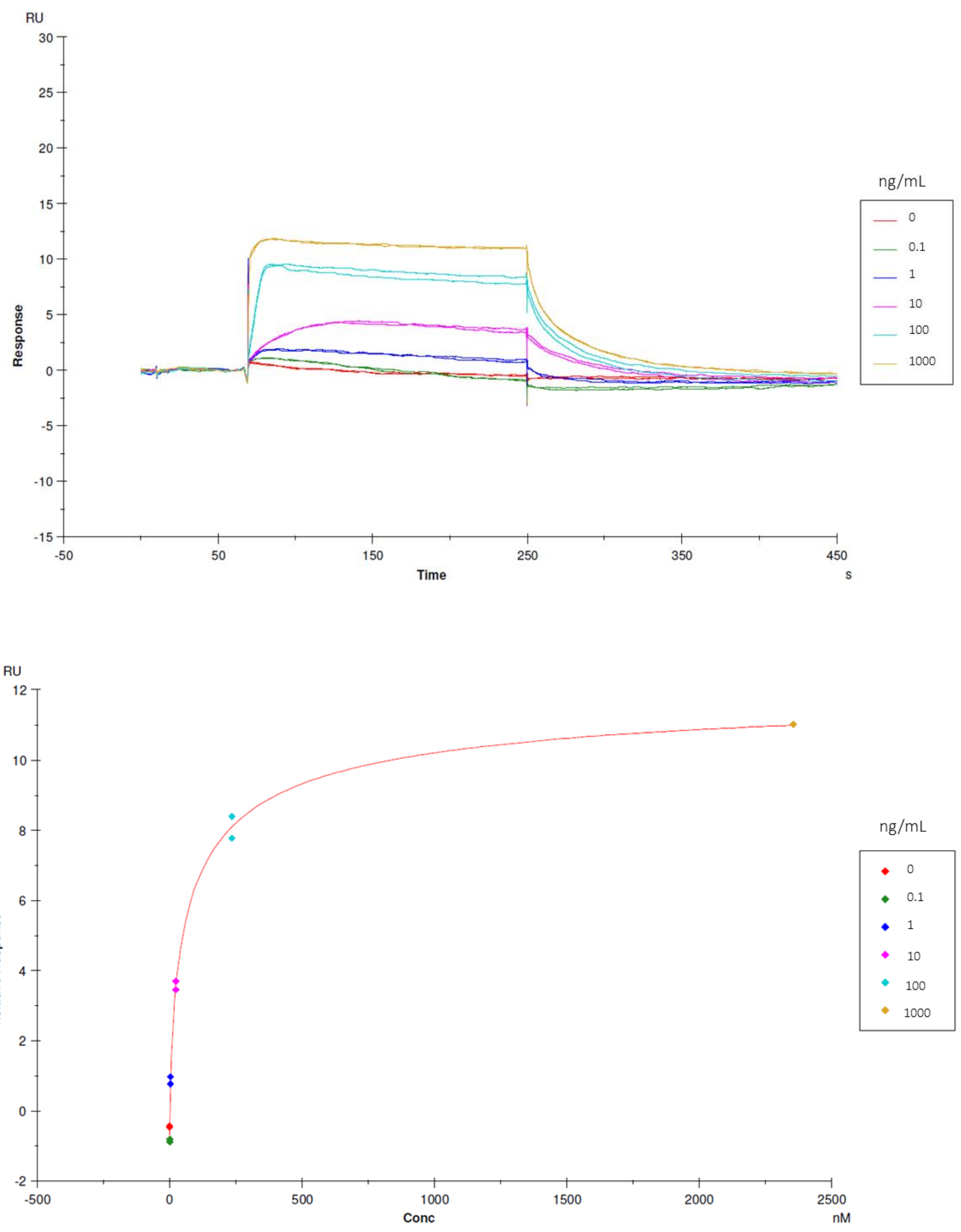

Figure S12 | Biacore T-200 HT-2 detection. a, Sensorgrams of Biacore T-200 response to HT-2 for different concentrations (from 1000 to 0.1 ng/ml). **b,** Relative binding response yields a HT-2 detection limit~1 nm/ml in Biacore T-200.

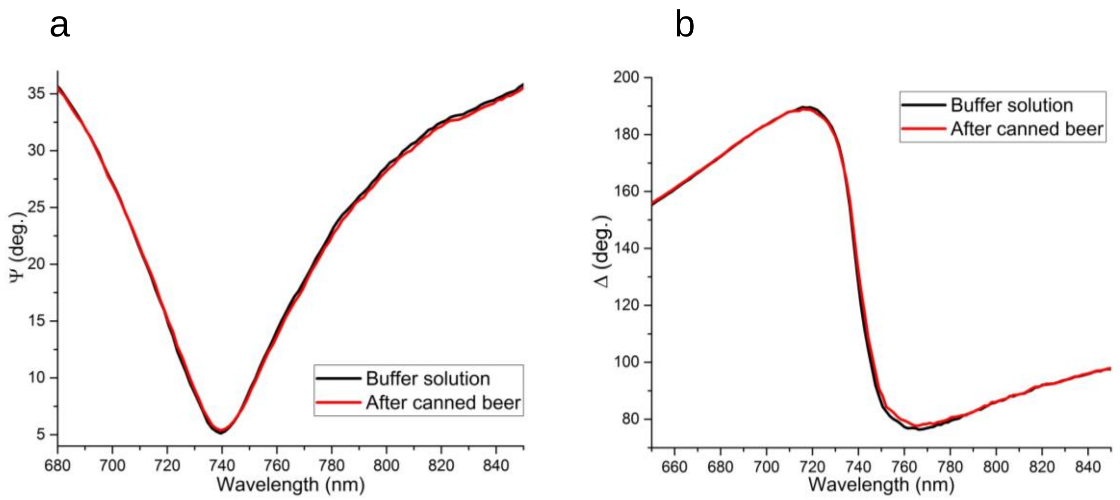

Figure S13 | HT-2 detection in a commercial beer. a, The SPR amplitude spectra measured in buffer before (the black curve) and after beer pumping (the red curve). **b,** The SPR phase spectra measured in buffer before (the black curve) and after beer pumping (the red curve).

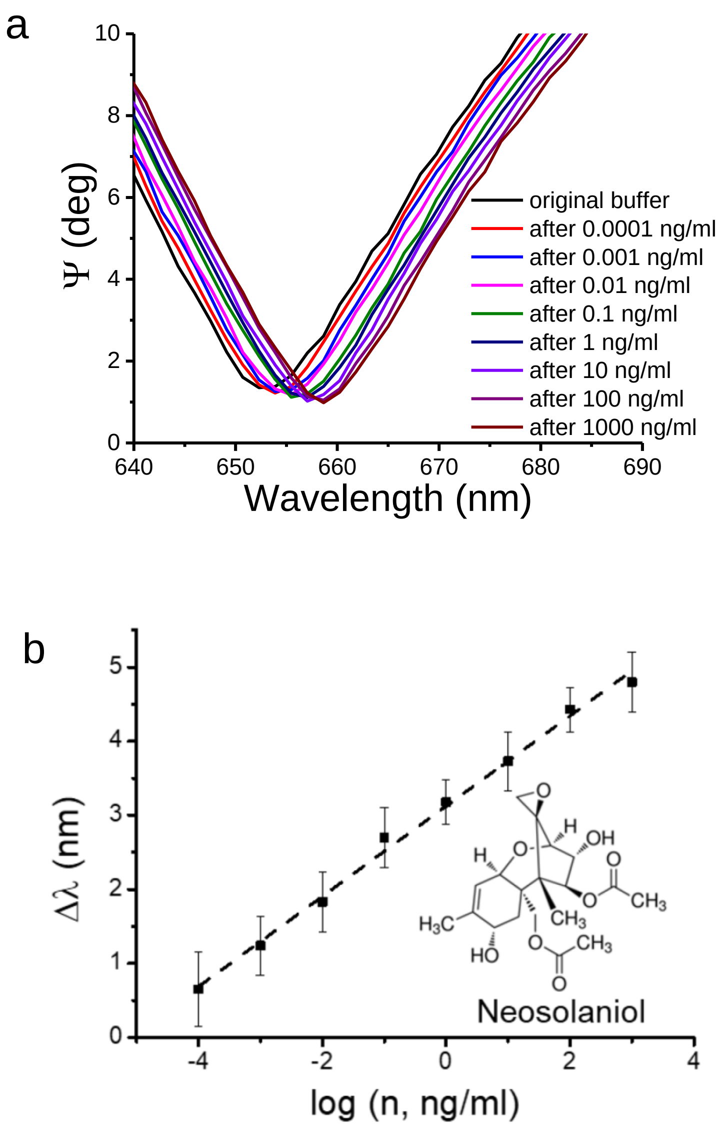

Figure S14 | Negative testing on SLG protected Cu SPR biosensor using neosolaniol. a, SPR curves of the biosensor after pumping different concentrations of neosolaniol. **b,** The shift of resonant wavelength (δλ) for Ψ as a function of concentration of neosolaniol. The inset shows the molecular structure of neosolaniol. The dark dashed line is the linear fit for δλ.
